# Exposure Risk of Chronic Wasting Disease in Humans

**DOI:** 10.3390/v12121454

**Published:** 2020-12-17

**Authors:** Satish K. Nemani, Jennifer L. Myskiw, Lise Lamoureux, Stephanie A. Booth, Valerie L. Sim

**Affiliations:** 1 Centre for Prions and Protein Folding Diseases, Edmonton, AB T6G 2R3, Canada; nemani@ualberta.ca; 2 Department of Medicine, Division of Neurology, University of Alberta, Edmonton, AB T6G 2R3, Canada; 3 Zoonotic Diseases and Special Pathogens, Public Health Agency of Canada, National Microbiology Laboratory, Winnipeg, MB R3E 3R2, Canada; jennifer.myskiw@canada.ca (J.L.M.); lise.lamoureux@canada.ca (L.L.); stephanie.booth@canada.ca (S.A.B.); 4 Department of Medical Microbiology and Infectious Diseases, Faculty of Health Sciences, University of Manitoba, Winnipeg, MB R3E 3R2, Canada

**Keywords:** chronic wasting disease (CWD), zoonotic potential, bovine spongiform encephalopathy (BSE), variant Creutzfeldt–Jakob disease (vCJD), sporadic CJD, CWD prions (PrP^CWD^), proteinase K resistant prion protein (PK-resPrP), strains, *PRNP* polymorphism, 129M/V polymorphism

## Abstract

The majority of human prion diseases are sporadic, but acquired disease can occur, as seen with variant Creutzfeldt–Jakob disease (vCJD) following consumption of bovine spongiform encephalopathy (BSE). With increasing rates of cervid chronic wasting disease (CWD), there is concern that a new form of human prion disease may arise. Currently, there is no evidence of transmission of CWD to humans, suggesting the presence of a strong species barrier; however, in vitro and in vivo studies on the zoonotic potential of CWD have yielded mixed results. The emergence of different CWD strains is also concerning, as different strains can have different abilities to cross species barriers. Given that venison consumption is common in areas where CWD rates are on the rise, increased rates of human exposure are inevitable. If CWD was to infect humans, it is unclear how it would present clinically; in vCJD, it was strain-typing of vCJD prions that proved the causal link to BSE. Therefore, the best way to screen for CWD in humans is to have thorough strain-typing of harvested cervids and human CJD cases so that we will be in a position to detect atypical strains or strain shifts within the human CJD population.

## 1. Introduction

Prion diseases are fatal transmissible neurodegenerative diseases thought to be caused by conformational conversion of cellular prion protein (PrP^C^) to pathological prion protein (PrP^D^) and its accumulation in both humans and animals [1]. In humans, prion diseases are categorized into three different forms: sporadic, genetic and acquired [2,3]. While sporadic Creutzfeldt–Jakob disease (sCJD) is the most prevalent form of human prion diseases, the acquired forms have generated a lot of fear, in particular over concern of zoonotic transmission through consumption of infected animals. The animal prion diseases comprise scrapie in small ruminants [1,4], bovine spongiform encephalopathy (BSE) in cattle [5], transmissible mink encephalopathy (TME) in mink [6], feline spongiform encephalopathy (FSE) in cats [7], spongiform encephalopathy in camels [8] and chronic wasting disease (CWD) in cervids [9].

Zoonotic transmission is a theoretical concern for all prion diseases, but, to date, the only documented transmission to humans has been from BSE-infected cattle. In the late 1980s, the outbreak of BSE (or mad cow disease) in cattle, and its transmission to humans through the food supply, resulted in a new form of prion disease, called variant CJD (vCJD), and caused 231 human deaths [10,11]. This has raised a concern for zoonotic transmission of other animal prion diseases, in particular CWD, as prevalence of this prion disease is rising.

CWD was first described in 1967 in a captive mule deer and later identified in black-tailed deer and Rocky Mountain elk in wild-life farms in Colorado and Wyoming [9,12]. In 2001, CWD was described in white-tailed deer in Nebraska and Dakota [13], and, in 2005, it was described in moose in Colorado [14]. Recently, CWD was also described in reindeer in Norway [15].

As the most infectious and contagious of all prion diseases, CWD is efficiently transmitted among cervids by both direct and environmental contacts [11]. Unlike BSE, CWD prions (PrP^CWD^) are distributed throughout the body of the diseased animal, including the peripheral and central nervous system, muscles, antler velvet and blood [16]. PrP^CWD^ is also shed in saliva, feces and urine [11,17] and can persist in the environment for many years, increasing exposure risk to all animal species within the ecosystem. This shedding, plus the fact that CWD occurs in wild migrating animals, makes its management more challenging than BSE, where banning specific risk material or contaminated feed was sufficient to reduce cases [18,19]. Attempts to control CWD spread include non-selective culling of the animals in endemic areas, regulations on the number of animals to be raised in a farm, guidance on carcass handling, mandatory testing of hunter harvested animals, feed bans, and prohibitions in importing cervids [20]. Despite these measures, CWD incidence continues to rise.

A major concern for human exposure to CWD comes through direct consumption of venison, but also consumption of other ruminants that might be reservoirs of adapted CWD prions [21,22]. Fortunately, there is a strong species barrier in most prion diseases, largely dependent on the degree of homology of PrP amino acid sequence between donor and recipient species. The barrier is not absolute though; it can be influenced by *PRNP* polymorphisms and different prion strains [23,24]. This is highly relevant, as studies are demonstrating the existence of several different CWD strains, each of which may have unique transmission properties towards humans.

As an added challenge, we do not know what the signs and symptoms of CWD would look like in humans. It might resemble sporadic CJD or present as something unlike any known human prion disease. In cervids, clinical signs of CWD include weight loss, isolation, and loss of fear towards humans. Polyuria, polydipsia, excessive drooling, ataxia and tremors are observed during the later stages of the disease [25,26]. Among cervids, the incubation period varies from 2 to 4 years [27]; if transmitted to humans, it could be decades.

In this article, we will review factors that could influence the transmission of CWD to humans, including risk of exposure, the influence of *PRNP* polymorphism and CWD strain on the species barrier, and transmission data from theoretical, experimental, and real-world scenarios. We will also present current surveillance data on human cases of prion disease and discuss how we might detect human presentations of CWD, should they arise.

## 2. Risk of Exposure

Whether humans will succumb to CWD transmission depends, in part, on the risk of humans encountering CWD in the real world. Human exposure to high levels of CWD prions is most likely to occur through direct handling of infected cervid carcasses or meat byproducts and through consumption of venison, velvet or other cervid byproducts [28]. Unlike BSE, where prions existed primarily in brain and spinal cord, prions in CWD are found at higher titers in tissues that are more highly consumed, such as skeletal muscle, and have been detected in cervids that are still sub-clinical and therefore more likely to be consumed [16,29]. Environmental exposure of humans to CWD prions is also possible, given that PrP^CWD^ is shed into the environment where it may remain infectious for decades. For these reasons, the prevalence of CWD and its geographical footprint are important factors.

Since the first description of CWD in the 1960s, there has been increasing geographic spread, with CWD now found in 26 states in the U.S., three provinces in Canada, South Korea, Norway, Sweden, and Finland [27,30]. In North America, the prevalence of CWD is variable, affecting up to 30% of free ranging animals in some areas, and as many as 80–90% of animals in captivity [26,27,31]. In Wisconsin, where prevalence of CWD is among the highest in the world, CWD cases in white-tailed deer doubled between 2010 and 2016, and male deer populations had prevalence of infection up to 40–50% with females at 20–30% [32]

## 3. *PRNP* Polymorphisms and Susceptibility

After exposure risk, we need to consider how efficiently CWD can cross the species barrier into humans. The transmission barrier is dependent on the amino acid sequence of host PrP^C^ and incoming misfolded PrP^Sc^ [24,33]. Therefore, polymorphisms of host PrP^C^ can play a major role in susceptibility. In humans, one polymorphism plays the greatest role in susceptibility; for cervids, 16 polymorphisms have been reported, with five linked to susceptibility in some studies [34].

### 3.1. Humans

In humans, the codon 129 M/V polymorphism influences both prion disease susceptibility and phenotype. In sporadic CJD, all polymorphisms are susceptible to disease, but homozygosity is greatly overrepresented, despite 129MV having a prevalence as high as 51% in some unaffected populations [24,35]. In contrast, vCJD has been seen almost exclusively in people with the 129MM genotype [36] with the exception of one case, possibly two, where vCJD occurred in association with the 129MV genotype [37]. The 129 M/V polymorphism also influences the type of prion conformation that is generated. Ninety percent of 129MM patients have Type 1, which is defined by a 21 kDa fragment after proteinase K (PK) digestion, whereas 80% of patients with 129VV or 129MV develop Type 2, which has a 19 kDa fragment after digestion [3,38,39].

### 3.2. Elk

The 132 M/L polymorphism in elk is of great interest as it corresponds to 129 M/V polymorphism in humans and may have a similar influence on susceptibility. Some studies have found a predominance of the 132M allele in infected elk, suggesting a predisposition of 132MM towards CWD development [40,41]. A small study that inoculated elk calves harboring all three genotypes (2 132MM, 2 132ML, 4 132LL) with 132MM CWD-infected elk brain inoculum found a dose-dependent effect of the M allele, with 132MM elk developing clinical signs at 23 months post infection (mpi), 132ML at 40 mpi [42] and 132LL at 59 mpi. In the 132LL animals, a novel PrP^D^ was detected, one with a more C-terminal proteinase K cleavage site (residues 98–133 compared to 78 or 82) [43]. This suggests that 132L may confer protection by prolonging the disease, at least when exposed to a mismatched inoculum containing 132MM CWD. The importance of strains used for these studies cannot be overstated. The premise of the species barrier relies on mismatched PrP sequence, so mismatched strain–host PrP would be predicted to have longer incubation periods. Further to this point, a larger study of 124 uninfected and 47 infected elk from Colorado found no effect of the 132M allele on susceptibility to CWD [44].

### 3.3. Mule Deer

Mule deer have two dimorphic polymorphisms, 225 S/F and 20 D/G, that have been associated with CWD susceptibility [34]. A study of 1482 free ranging mule deer in Colorado and Wyoming, including 289 CWD-infected animals, found that mule deer with the 225SS allele were 30 times more likely to have CWD compared to those with 225SF [45]. This suggests a potentially protective effect of the 225F allele, analogous to the 132L allele in elk. Interestingly, there was no influence of the 20 D/G polymorphism in these animals [45], whereas in an identical but smaller study of 249 mule deer in Canada, the presence of the 20G allele was associated with twice the rate of CWD infection compared to 20DD [46]. Whether these differences reflect sample size or exposure to different CWD strains is unclear. It is also intriguing to consider how changes in codon 20 could contribute to altered susceptibility, given that it lies outside the sequence included in the mature form of PrP.

### 3.4. White-Tailed Deer

In white-tailed deer, the *PRNP* alleles Q95/G96 (wild type), Q95/S96 (referred to as S96), H95/G96 (referred to as H95), and H95/S96 are associated with variable CWD susceptibility [47,48]. When white-tailed deer containing these polymorphisms were infected with wild-type hunter-harvested deer from Wisconsin, infection was fastest in the wild-type Q95/G96 deer at 693 days post-infection (dpi), followed by 956 dpi in S96 deer, 1508 dpi in H95 deer, and 1596 dpi in H95/S96 deer, suggesting that S96 and H95 alleles in the host led to longer incubation time and partial protection [49]. Inoculation of PrP^CWD^ from 96SS white-tailed deer into transgenic mice harboring alleles 96GG, 96GS and 96SS resulted in disease in 96GG mice, delayed disease in 96GS mice, and no disease in the 96SS mice. This further supported the hypothesis that the S allele is protective or can slow down the disease [50]. One other polymorphism has been recently discovered, 116A/G, but its role in CWD susceptibility has not yet been studied in detail [51].

### 3.5. Moose

Two polymorphisms, 109 K/Q and 209 M/I, have been identified in moose *PRNP*. Both 109K/209M and 109K/209I are found in North America whereas 109Q/209M and 109K/209M alleles are found in European moose [52]. However, association of *PRNP* polymorphism with CWD susceptibility has not yet been demonstrated experimentally or naturally in moose, because of low numbers [14,53,54].

### 3.6. Reindeer

In reindeer, there are polymorphisms at several sites: 2 V/M, 129 G/S, 138 S/N, 169 V/M, 176 N/D and 225 S/Y. Notably, 176DD is seen only in reindeer [52]. The heterozygous 138SN is associated with delay or resistance to CWD infection during oral transmission [55], but intracranial inoculation of 138SN reindeer does result in CWD infection [56].

## 4. The Role of Strains and Adaptation

In addition to the primary sequences of PrP, transmission is also dependent on strain [33]. Strains are defined by their abilities to cause distinct clinical signs, incubation periods, pathological profiles and/or PrP biochemical characteristics that are retained on passage [24,33,57]. They are thought to arise from different conformations of misfolded PrP, which may or may not have the same underlying PrP sequence. The ability of strains to propagate and cause disease may vary within the same host and a subset of strains may cross the species barrier and cause disease by adaptation after passage in different host [24]. For example, the inoculation of sheep-passaged bovine prions into Tg mice that express cervid PrP caused prion infection, whereas directly infecting these mice with bovine prions did not [58]. Such species adaptation through passage is of high relevance to CWD because PrP^CWD^ exists in the environment, available for other host infections.

There is evidence for different strains of CWD, often emerging through passage in transgenic mice, but the strains themselves are not yet well characterized and are influenced heavily by PrP polymorphisms. Whether all the strains detected across the cervids are different is not clear because there are few ways to discriminate subtle differences in strains.

### 4.1. Elk

When Tg(CerPrP)1536+/− mice that express cervid PrP were inoculated with elk PrP^CWD^, two strains, CWD 1 and CWD 2, were produced, whereas inoculation with mule deer PrP^CWD^ produced strain mixtures [59]. Tg12 mice that express elk 132M PrP were inoculated with elk PrP^CWD^ of genotype 132MM, 132ML or 132LL and also produced two different PrP^D^ profiles, dependent on the polymorphism of the inoculum [60].

### 4.2. Deer

Oral inoculation of Wisc-1 CWD into white-tail deer harboring wt/wt, wt/S96, H95/wt, or H95/S96 PrP led to the detection of Wisc-1 strain in wt/wt and wt/S96 but the presence of new strain H95+ in H95/wt and H95/S96 [47,48].

### 4.3. Moose

Two types of naturally occurring CWD have been found in moose: 209MM in Norway and 209II in Canada, each with distinct PrP^D^ profiles and neuropathology [54].

Given the growing evidence for strains and strain mixtures within naturally infected cervids, and the implications this has for transmission, more in vitro and in vivo studies of different CWD isolates and different PrP polymorphisms are necessary to fully assess the risks of CWD adaptation and potential to cross species barriers.

## 5. Crossing the Species Barrier

To date, there are no reports of prion disease transmission to livestock or humans in CWD endemic areas, either because there is a strong species barrier to non-cervid species [61], or not enough time has passed for sufficient dose exposure or incubation period. Epidemiological data, as well as in vivo and in vitro experiments, have been used to assess the likelihood of CWD crossing the species barrier into humans, with mixed results.

### 5.1. In Vitro Studies

Research groups have used protein misfolding cyclic amplification (PMCA) or real-time quaking-induced conversion (RT-QuIC) to test whether CWD prions can propagate in the presence of PrP^C^ from other species, including human. In a PMCA study of 12 non-cervid species, ferrets, voles, hamsters and field mice supported the conversion whereas prairie dog, Tg huPrP mice, coyote, cat, macaques and wild-type mice failed to support amplification [22,62]. Analysis of PrP sequences in the β2–α2 (166–175) loop suggested that species containing asparagine at position 170 supported conversion, in addition to the ferret which has serine at position 170 but still was able to amplify CWD prions. It is interesting to note that, except for ferret, all other non-cervid species possessed phenylalanine at residue 175, whereas ferret has leucine at this site, which may induce stability in the loop and thereby support conversion [22,62].

Other studies have successfully generated protease-resistant human PrP aggregates after exposure to PrP^CWD^. Human brain homogenates, transgenic mice, and 239F cells expressing human PrP with the 129M or 129V polymorphism were able to support PrP^CWD^ amplification in PMCA, with preferential conversion of PrP harboring the 129M allele. Interestingly, the number of amplification cycles required differed depending on the strain of CWD used; mule deer PrP^CWD^ required multiple rounds, whereas elk and white-tailed deer PrP^CWD^ required only one [61,63,64]. Whether this represents a difference in species barrier is unclear. RT-QuIC has also been used to demonstrate the ability of PrP^CWD^ to seed the aggregation of human PrP [65].

### 5.2. In Vivo Experiments

Attempts to transmit CWD strains into Tg mice expressing human PrP with different polymorphisms 129MM, MV and VV have failed to induce any disease [66,67]. Intracerebral and oral inoculation of cervid prions into squirrel monkeys did result in PrP^D^ deposition and spongiform degeneration [68,69], but intracerebral and oral inoculation into macaques, which are more closely related to humans, failed to cause any signs of disease [69].

When interpreting transmission studies, it is important to consider the species, method and route of infection used. For example, intracerebral inoculation of cattle and sheep with PrP^CWD^ from mule deer resulted in disease within 3–5 years for cattle and 3 years for sheep [70,71]; however, oral inoculation of mule deer PrP^CWD^ into 12 calves failed to cause any disease [72]. Given that oral exposure is the most probable method of real-world transmission to humans and other species, the fact that recurrent sonication in vitro or intracerebral inoculation in vivo can induce aggregation or disease does not necessarily predict the future CWD landscape.

### 5.3. Human Data

There are no documented case reports of CWD transmission to humans. The largest exposure occurred at a sportsman feast in Oneida county, NY, where 200 people were unknowingly exposed to CWD-infected venison meat. No cases of CJD were reported after 6 years of follow-up with 81 participants [73]. Other reports have tried to link cases of CJD with venison exposure, but proof of exposure to CWD is lacking in most cases. From 1997 to 1990, there were reports on the deaths of three unusually young people diagnosed with CJD who had a history of regular consumption of deer or elk meat [74]. Subsequently there were reports on the diagnosis of CJD in hunters who participated in wild game feast in Wisconsin (CDC 2003 report), detection of CJD in Colorado in a 52-year-old woman who had a CWD laboratory exposure, and a 25-year-old man with CJD who had consumed venison meat in a CWD endemic area [75]. In all the above studies, patients had *PRNP* genotypes, PrP^D^ biochemical characteristics and pathologies suggestive of sporadic CJD. A subsequent study from Colorado demonstrated no statistically significant increase in CJD cases within CWD-endemic areas [76].

## 6. Detecting CWD in Humans; the Role of Surveillance and Strain Typing

If transmission of CWD to humans occurs, it will most likely be through the oral route and occur in a CWD-endemic area where cervids are hunted and/or consumed [19]. We predict that it would induce a neurological phenotype, but whether it would be clinically or biochemically distinct from sCJD is impossible to know. In a PMCA study where elkPrP^CWD^ generated PrP^res^ from human brain homogenate harboring 129M PrP^C^, the resultant huPrP^res^ was identical to sCJDMM1 [77]. Hopefully, from a surveillance perspective, any CWD presenting in humans will have a unique clinical presentation and/or a PrP^D^ biochemical profile that matches that of a known CWD strain, analogous to the PrP^BSE^ fingerprint seen in vCJD. Of course, to identify the emergence of a new PrP^D^ profile, we require strain typing on all CWD and CJD strains that are currently occurring. If a unique fingerprint or strain shift does not occur, we will still have surveillance to detect any increased incidence of CJD cases among patients consuming venison in CWD-endemic areas, recognizing that such exposures may take decades or more to declare themselves.

**Figure 1:**
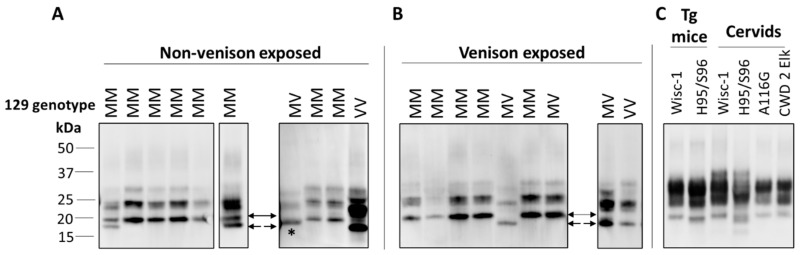
Immunoblot profiles of PK-resistant PrP from brains of Creutzfeldt–Jakob disease (CJD) patients, with or without exposure to venison, compared with chronic wasting disease (CWD) strains. (**A**,**B**) Typing studies for non-venison (**A**) and venison (**B**) exposed CJD cases were carried out as described previously [78], with 10% brain homogenates in lysis buffer (LB100) (100 mM Tris HCl pH 7.0, 100 mM NaCl, 10 mM EDTA, 0.5% Nonidet-P 40, 0.5% sodium deoxycholate) digested with 70 U/mL PK for 1 h at 37 °C. Different sample volumes were loaded or blots from different runs were included to obtain a similar PrP signal intensities for comparison of banding profiles. Type 1 (21 kDa) is indicated by a solid arrow, Type 2 (19 kDa) by dashed arrow, and 20 kDa from variably protease sensitive prionopathy (VPSPr) indicated with *. Primary Ab: 3F4. (**C**) Typing study of CWD strains, with 10% homogenates in LB100 pH 8.0 digested with 30 U/mL PK. Wisc-1 was propagated in Tg33 mice and H95/S96 was first passaged in Tg60 mice. BH from deer and elk harboring different polymorphisms was also analyzed. Primary Ab: Sha31

**Figure 2:**
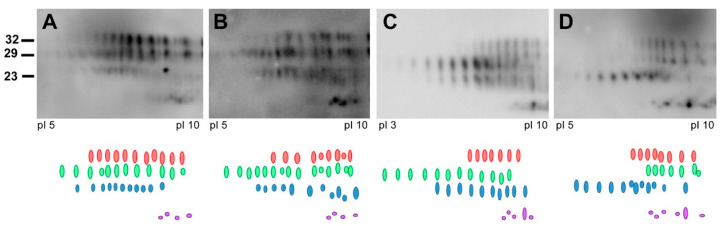
Representative images of PrP^Sc^ 2D-electrophoresis are shown. PrP^Sc^ was precipitated from brain homogenate using sarkosyl and NaPTA. Extracted proteins were separated in the first dimension on Ready-Strip IPC strips (Bio-Rad) and a pH gradient of 3–10. Following equilibration in SDS and reducing/alkylating agents, the second dimension of the gel was performed on Criterion Tris-HCL polyacrylamide precast gels (8–16%) prior to Western blotting. PrP^Sc^ was detected using the 3F4 antibody and an HRP secondary. Four strips of spots were identified at different molecular weights: 32 kDa (red), 29 kDa (green), 23k Da (blue) and 13/14 kDa (purple). (**A**) Non-venison exposed CJD subject; (**B**–**D**) venison exposed CJD subjects.

In ongoing surveillance in Canada, where cases of CJD are identified and flagged for a history of venison consumption, we have not yet seen a change in CJD strain profiles in CWD-affected provinces. Several parameters have been examined as part of this strain typing and comparison, including clinical presentations, MRI findings, pathological profiles and pattern of PrP^D^ resistance to PK (Figure 1). Additionally, analysis of total PrP^D^ by two-dimensional gel electrophoresis, which detects differences in the extent of glycosylation and other posttranslational modifications that cannot be detected by one-dimensional blotting, also has not revealed any differences in CJD strain patterns between non-exposed and venison-exposed cases (Figure 2). This is perhaps not surprising, given that human exposure to CWD so far has been low, and incubation periods may be very long. However, this analysis provides the most in-depth profile of the current human CJD strain landscape.

**Figure 3:**
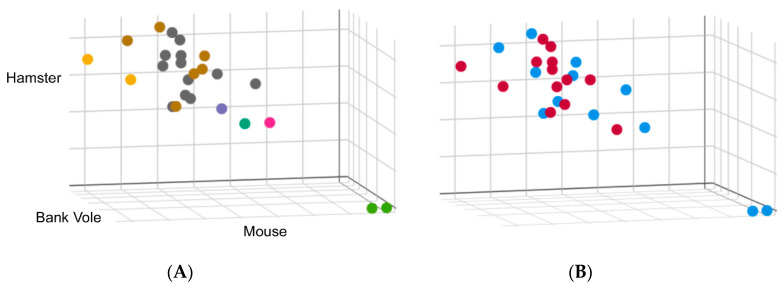
Real-time quaking-induced conversion (RT-QuIC) was performed on 10^−4^ dilutions of brain homogenate from 25 cases of sCJD using 3 different substrates: full-length mouse, hamster and bank vole. Conversion rate was measured by comparing the time at which fluorescence in a given reaction exceeds a pre-defined threshold, i.e., the lag phase. Lag phases for each case were normalized to a 263 K hamster brain homogenate run concurrently as a standard to allow direct comparison between each run. These values were plotted on a scatter plot where the axes represent the normalized lag phase for each substrate. (**A**) Cases were colored according to the biochemical subtypes: Type 1, shown in grey, and Type 2, shown in brown. Two cases where a mixture of Type 1 and Type 2 were identified are shown in yellow. Cases shown in green are two cases of variably protease sensitive prionopathy (VPSPr). A Type 1 case with a biochemically distinct denaturation profile is shown in green and two others that do not exhibit typical glycoform patterns by Western blot shown in pink and purple. (**B**) Data were colored to reflect RT-QuIC seeding from brains of CJD patients, with or without exposure to venison, shown as blue or red, respectively.

We also need data on the growing CWD strain landscape. Currently, immunohistochemistry, enzyme immunoassay, Western blotting, RT-QuIC and PMCA are the methods available for detection of PrP^CWD^ [61]. Unfortunately, most strain information is obtained from immunohistochemistry and Western blotting and levels of PrP^CWD^ may be too low to detect by these methods unless animals are at a late stage of the disease. PMCA and RT-QuIC can detect low levels of PrP^CWD^ in the preclinical phase or subclinical phase, but have traditionally been used for detection, not strain typing. We have recently found that the kinetics of RT-QuIC may have value as a CJD surveillance tool, as we detected remarkably consistent kinetic seeding profiles for distinct CJD types, allowing for the identification of a number of outliers (Figure 3). Interestingly, the outliers identified through RT-QuIC correlated with rare subtypes identified by Western blot analysis and pathology. These findings suggest that RT-QuIC may be useful in differentiating some human prion strains.

As we gain more insight into CWD strains and their properties, we need to continue monitoring and strain-typing human cases, looking for evidence of new PrP fingerprints. We must also watch for increasing CJD cases in CWD-endemic areas. On one hand, evidence supports a strong species barrier to human transmission of CWD. On the other hand, we are identifying a variety of CWD strains, some of which can convert human PrP under experimental conditions. Humans will also co-exist with PrP^CWD^ in the environment for decades to come. Will these factors be sufficient to allow strain adaptation and a crossing of the species barrier? Only time will tell.
